# Single Image Haze Removal via Multiple Variational Constraints for Vision Sensor Enhancement

**DOI:** 10.3390/s25237198

**Published:** 2025-11-25

**Authors:** Yuxue Feng, Weijia Zhao, Luyao Wang, Hongyu Liu, Yuxiao Li, Yun Liu

**Affiliations:** 1 College of Sericulture, Textile and Biomass Sciences, Southwest University, Chongqing 400715, China; 2 Faculty of Innovation and Design, City University of Macau, Macau SAR, China; 3 College of Artificial Intelligence, Southwest University, Chongqing 400715, China

**Keywords:** vision sensors, haze removal, atmospheric scattering model, mixed variational model, scene recovery

## Abstract

Images captured by vision sensors in outdoor environments often suffer from haze-induced degradations, including blurred details, faded colors, and reduced visibility, which severely impair the performance of sensing and perception systems. To address this issue, we propose a haze-removal algorithm for hazy images using multiple variational constraints. Based on the classic atmospheric scattering model, a mixed variational framework is presented that incorporates three regularization terms for the transmission map and scene radiance. Concretely, an $\ell_{p}$ norm and an $\ell_{2}$ norm were constructed to jointly enforce the transmissions for smoothing the details and preserving the structures, and a weighted $\ell_{1}$ norm was devised to constrain the scene radiance for suppressing the noises. Furthermore, our devised weight function takes into account both the local variances and the gradients of the scene radiance, which adaptively perceives the textures and structures and controls the smoothness in the process of image restoration. To address the mixed variational model, a re-weighted least square strategy was employed to iteratively solve two separated subproblems. Finally, a gamma correction was applied to adjust the overall brightness, yielding the final recovered result. Extensive comparisons with state-of-the-art methods demonstrated that our proposed algorithm produces visually satisfactory results with a superior clarity and vibrant colors. In addition, our proposed algorithm demonstrated a superior generalization to diverse degradation scenarios, including low-light and remote sensing hazy images, and it effectively improved the performance of high-level vision tasks.

## 1. Introduction

Haze or fog is a common atmospheric phenomenon that frequently degrades the performance of imaging sensors in outdoor environments. Under hazy conditions, images and videos captured by camera-based sensing systems suffer from severe degradation due to light scattering caused by suspended particles in the atmosphere. This scattering leads to blurred scene details, faded colors, and reduced saturation, which collectively diminish the image contrast, visibility, and color integrity [1], as well as distort the color perception [2]. To be specific, haze-induced degradations exhibit different characteristics across image types in a dataset: urban scenes often suffer from strong color fading and contrast loss due to dense haze and artificial illumination; rural and mountain scenes exhibit heavy blurring and reduced visibility caused by thick atmospheric scattering over long distances; and waterbody and vegetation-rich scenes tend to show severe desaturation and color shifts owing to light absorption and reflection by moisture and aerosols. These haze-induced degradations produce low-quality visual data that obscure scene structures and textures, posing significant challenges for vision-based sensing applications, including object detection, recognition, and tracking, which rely on clear visual inputs. Therefore, enhancing the image quality under haze conditions is essential to improve the robustness and reliability of sensor-based vision systems in real-world scenarios such as autonomous driving, surveillance, and environmental monitoring.

To improve the image quality, some fusion-based methods [3,4,5] have been proposed for haze removal. Unfortunately, they have failed to consider the physical mechanism, which results in incomplete dehazing. In order to describe the formation of a hazy image, the classic atmospheric scattering model (ASM) [6,7,8] is widely employed, which illustrates that a hazy image is a linear combination of attenuated scene radiance and ambient airlight. By inverting this model, image recovery is achieved, resulting in a clear image. Unfortunately, directly inverting the ASM is an ill-posed problem due to the presence of two unknown parameters, which makes it difficult to recover the scene radiance from a single input image. To address this challenge, researchers have proposed several effective priors [9,10,11,12,13,14,15,16,17,18] to estimate the scene transmission. These hand-designed priors are primarily based on statistical observations or human experience; however, they do not always hold true, making them difficult to apply in complex haze scenarios. For example, the famous dark channel prior [9] may not perform effectively in large sky regions, resulting in color distortion in the processed results.

Thanks to the continuous advancements in graphics processing units (GPUs), deep learning techniques have been successfully applied to various fields of computer vision, such as image fusion [19,20], image classification [21,22], etc. Numerous effective networks [23,24,25,26,27,28,29,30,31,32,33,34,35,36,37,38] have been presented to improve the visual quality of hazy images. These learning-based methods typically rely on complex network architectures that learn useful features from large-scale image datasets to remove haze. Early approaches [23,24,25] mainly leveraged convolutional neural networks (CNNs) for feature extraction to estimate the medium transmission and subsequently rely on the ASM to recover clear images. However, the inaccurate estimation of transmission, due to the inherent uncertainty of deep learning models, may result in an insufficient dehazing ability. Therefore, more recent architectures [27,28,29,30,31,32,33,34], based on generative adversarial networks (GANs) [39] and transformers [40], have been developed to perform direct end-to-end mapping from hazy images to clear results. While these learning-based methods have demonstrated an impressive performance on synthetic hazy images, they often struggle to generalize in real-world haze scenarios. As pointed out in [41], this is primarily due to the substantial feature and domain discrepancies between real and synthetic data, which limit their effectiveness in practical applications.

To overcome the above limitations, variation-based strategies [42,43,44,45,46,47,48,49,50] have been employed to combine the ASM within an optimization framework, with the aim of improving the perceptual quality of hazy images. These approaches typically leverage various types of regularization terms, such as the $\ell_{2}$, $\ell_{1}$, and $\ell_{0}$ norms, to impose constraints on the medium transmission and scene radiance, thereby promoting smoothness, sparsity, or edge preservation. The $\ell_{2}$ norm is commonly used as a data term to enforce consistency between the observed hazy image and the estimated parameters, while the $\ell_{1}$ norm encourages sparsity in the estimated transmission, allowing for the preservation of important details. In contrast, the $\ell_{0}$ norm is employed to enforce a piecewise-constant structure, effectively preserving sharp edges and discontinuities. Although these variational dehazing methods that use integer norms have proven effective in several cases, they lack the necessary flexibility, often struggling with complex scene structures, which limits their ability to produce high-quality dehazed images.

In this paper, we propose a novel mixed variational framework to improve the quality of hazy images. Our proposed framework includes three regularization terms: a flexible $\ell_{p}$ norm and an $\ell_{2}$ norm to constrain the medium transmission, and a weighted $\ell_{1}$ norm to enforce the scene radiance. Different from existing variation-based methods, our mixed variational framework integrates multiple constraints, particularly the flexible $\ell_{p}$ norm, which effectively preserves the edges and smooths the details in the estimated transmission map, allowing it to adapt to complex haze scenarios. In addition, we designed a content-aware weight function that considers both the local variances and the gradients of the scene radiance, which helps to regulate the smoothness and prevent noise interference in the recovered result. To compensate for the brightness, a gamma correction was applied to the recovered results in order to obtain the final result. Qualitative and quantitative comparisons on both synthetic and real hazy scenes demonstrated that our proposed algorithm outperformed the other dehazing techniques, producing visually appealing results with fine details, vivid colors, a high contrast, and a well-balanced brightness.

The main contributions of our work can be summarized as follows:

- We propose a novel variational dehazing framework that incorporates multiple constraints: a flexible $\ell_{p}$ norm, an $\ell_{2}$ norm, and a weighted $\ell_{1}$ norm. The framework simultaneously estimates the accurate transmission map and produces high-quality clear results. Compared to previous methods based on integer-order norms, our embedded $\ell_{p}$ regularization offers a greater flexibility, making it more adaptable to a wide range of haze scenarios.
- We designed a weight function that incorporates both the local variances and the gradients of the clear image, which effectively controls the smoothness of the recovered image, helping to suppress noise and preserve important details.
- Experiments conducted on both synthetic and real hazy data demonstrated the competitive performance of our proposed algorithm in terms of the image quality and objective metrics.

The remainder of this paper is organized as follows. Section 2 reviews related work on image-dehazing methods. Section 3 presents the proposed mixed variational framework along with the associated optimization procedure. Section 4 describes the experimental setup, provides comparisons with state-of-the-art methods, and evaluates the generalization on remote sensing and low-light hazy images, followed by the limitations and discussions. Finally, Section 5 concludes the paper.

## 2. Related Work

In this section, we provide a review of several recent dehazing algorithms, including prior-based methods, learning-based methods, and variation-based methods.

### 2.1. Prior-Based Dehazing Methods

Prior-based dehazing methods usually rely on an atmospheric scattering model and propose hand-crafted priors to estimate the model parameters for recovering the scene radiance. The most famous method of this type is the dark channel prior (DCP) [9] proposed by He et al. The DCP [9] reveals that at least one color channel in non-sky regions of clear images includes some pixels with a low intensity, even approaching zero. On the basis of the DCP, the transmission and the atmospheric light are estimated for model inversion. Several DCP-based variants [51,52] have been proposed to increase the quality of degraded images. However, the DCP may be ineffective in image regions that are similar to the atmospheric light, such as sky areas. Furthermore, DCP-based dehazing approaches may lead to overly dark results. Subsequently, several effective priors were proposed to estimate the model parameters. For instance, Zhu et al. [10] developed a color attenuation prior (CAP) to model a linear relationship between the scene depth and both the brightness and saturation. A non-local prior (NLP) [11] was proposed to form the haze lines for recovering the transmission map. Bui et al. [12] constructed a color ellipsoid prior (CEP) to compute the transmissions via color ellipsoid geometry. A gradient profile prior (GPP) [13] was designed to estimate the depth map from the input hazy image. Ju et al. [14] employed a global-wise strategy and proposed a gamma correction prior (GCP) to recover the scene albedo. From the perspective of a region-wise strategy, Ju et al. [15] devised a region line prior (RLP) and a 2D joint optimization function to restore the haze image. Ling et al. [16] proposed a saturation line prior (SLP) and transformed the transmission estimation into the construction of saturation lines. More recently, heterogeneous priors [17] were developed to estimate the transmission and atmospheric light. An ambient light similarity prior (ALSP) [18] was developed to estimate the scene transmission for fast scene recovery. These above prior-based dehazing methods can restore the hazy image and improve the visual quality to some degree. Unfortunately, these presented hand-designed priors are not always applicable to complex real-world haze scenarios.

### 2.2. Learning-Based Dehazing Methods

Depending on the powerful ability of feature extraction, learning-based networks have been gradually developed to achieve single image quality improvements under haze conditions. DehazeNet [23] was first proposed to estimate the medium transmission map from an input hazy image. Ren et al. [24] proposed a multi-scale convolution neural network (MSCNN) to build the relationship between a hazy image and a transmission map. Furthermore, Ren et al. [25] leveraged the holistic edge-guided network to refine the transmission map. An all-in-one dehazing network (AOD-Net) [26] was designed to estimate one key parameter that unifies transmission and atmospheric light into a single formula. Using generative adversarial networks (GANs) [39], Deng et al. [27] proposed a haze-aware representation distillation GAN (HardGAN) to achieve haze removal. Dehaze-AGGAN [28] exploits an enhanced attention-guide GAN to overcome the dehazing issue of remote sensing images. Zhang et al. [29] leveraged generative adversarial and self-supervised learning to obtain a natural clear image. A GAN-based prior-guided dehazing network [30] was developed to transfer various priors learned from clean data to haze removal. In recent years, a transformer [40] consisting of an encoder and a decoder has been applied to image-dehazing tasks. Guo et al. [31] integrated a CNN and a transformer and proposed a transmission-aware 3D position embedding module. DehazeFormer [53], as an improvement of the Swin Transformer [32], is presented to remove the non-homogeneous haze. Liu et al. [33] proposed NightHazeFormer to overcome multiple degradations of nighttime hazy images. SelfPromer [34] adopts the prompt based on the depth difference between the input hazy images and clear images to enhance the dehazing performance. KA-Net [54] introduces a localization- and removal-based network architecture to improve the adaptability in real-world scenarios. Although these above dehazing networks can improve the visual quality for synthetic hazy images, they are unable to generalize well for real data. Therefore, learning-based dehazing methods may not be applicable to real hazy scenarios. Furthermore, they also consume extensive hardware resources and require a large amount of training data and time.

### 2.3. Variation-Based Dehazing Methods

Variation-based strategies have been successfully applied in various computer vision tasks, such as medical image segmentation [55,56] and deraining [57,58]. Therefore, variation-based dehazing methods have gradually been developed to enhance the visual quality of hazy images. For example, Meng et al. [42] explored the inherent boundary constraint and constructed a weighted $\ell_{1}$-norm regularization to estimate the transmission map. Galdran et al. [43] exploited the gray-world hypothesis to extend a perception-inspired variational model to realize the contrast enhancement of hazy images. In [44], Wang et al. combined the Retinex assumption and Koschmieder’s law to construct a constrained total variation model for haze removal. Afterwards, non-local total variation regularization [45] was proposed to preserve the depth-aware structures. Liu et al. [46] presented a unified variational model consisting of two $\ell_{1}$-norm regularization terms to estimate the transmission map and clear image. Recently, Liu et al. [47] employed an $\ell_{0}$-norm regularization constraint to enforce the reflectance component for achieving nighttime image dehazing. Jin et al. [48] combined the total generalized variation (TGV) and total variation to respectively constrain the scene depth and haze-free image. Li et al. [49] exploited the Gaussian total variation (GTV) [59] to acquire the transmission map. Compared to prior-based and learning-based methods, variational dehazing models not only offer flexibility and interpretability for more complex real-world scenarios, but they also require fewer computational resources and less data. Unfortunately, the existing variation-based dehazing methods typically adopt integer-order norms, such as $\ell_{0}$, $\ell_{1}$, and $\ell_{2}$, to construct the regularization terms, which fail to achieve an accurate estimation of the model parameters, resulting in a poor dehazing performance. Although some variational models [50,60,61] have been developed to design the $\ell_{p}$-norm constraint for nighttime low-quality enhancement, they are difficult to directly apply for image quality improvements under haze conditions.

## 3. Methodology

**Figure 1:**
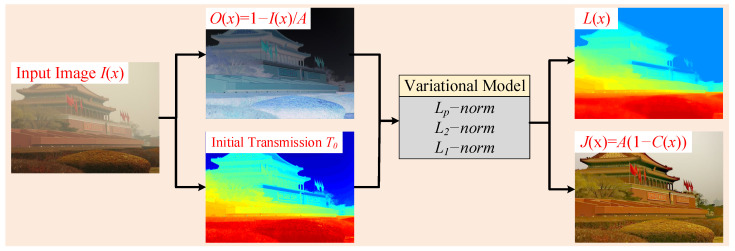
Flowchart of our proposed framework.

In this section, the classic ASM and its transformation are first described. Then, our proposed mixed variational model is developed. At last, the numerical solver and algorithm procedure of our proposed variational model is illustrated in detail. An overview of the proposed algorithm is presented in Figure 1.

### 3.1. Atmospheric Scattering Model (ASM)

Under haze conditions, the light in the imaging path is scattered by the suspended particles in the atmosphere, leading to the attenuation of the reflected light from the scene. Meanwhile, the scattered ambient light blends with the light received by the capturing device, leading to image degradation. In order to explain the degradation procedure, the ASM [6,7,8] is introduced to describe the generated hazy images:

(1) $$I\left(x\right)=J\left(x\right)t\left(x\right)+A\left(1-t\left(x\right)\right)$$

where *x* denotes the pixel location, and I(x) and J(x) represent the observed hazy image and the clear image, respectively. *A* stands for the global atmospheric light and t(x) represents the medium transmission, indicating the portion of light that reaches the capturing device.

By dividing by *A* on both sides of Equation (1), Equation (1) can be rewritten as follows:

(2) $$\left(1-\frac{I\left(x\right)}{A}\right)=\left(1-\frac{J\left(x\right)}{A}\right)t\left(x\right)$$

For convenience, we used the matrix form to rewrite Equation (2) as follows:

(3) O=C∘L

where “∘” denotes the Hadamard product, $O=1-\frac{I\left(x\right)}{A}$, $C=1-\frac{J\left(x\right)}{A}$, and $L=t\left(x\right)$. C=1−J(x)/A denotes the normalized inverted scene radiance relative to the atmospheric light *A*, while L=t(x) corresponds to the medium transmission, providing a physically interpretable link to the standard atmospheric scattering model. Since J(x), t(x), and *A* are unknown, recovering the haze-free image J(x) from an input single hazy image I(x) is an ill-posed problem that cannot be solved directly. Therefore, designing effective constraints helps in solving this problem. To achieve this goal, our work proposes a mixed variational model consisting of multiple effective constraints to simultaneously recover the medium transmission and the haze-free image.

### 3.2. Mixed Variational Model

In this subsection, a novel mixed variational model that simultaneously estimates the medium transmission *L* and the inverted scene radiance *C* from the inverted intensity of the input image *O* is formulated as follows:

(4) $$E\left(C,\;L\right)={∥C\circ L-O∥}_{2}^{2}+\lambda_{1}{∥\nabla L∥}_{p}+\lambda_{2}{∥L-T_{0}∥}_{2}^{2}+\lambda_{3}{∥W\circ \nabla C∥}_{1}$$

where $\lambda_{1}$, $\lambda_{2}$, and $\lambda_{3}$ are the weight parameters, which balance three regularization terms. ${∥\cdot ∥}_{p}$, ${∥\cdot ∥}_{2}$, and ${∥\cdot ∥}_{1}$ stand for $\ell_{p}$ norm, $\ell_{2}$ norm, and $\ell_{1}$ norm, respectively. ∇ is the first-order derivative operator. The first part, ${∥C\circ L-O∥}_{2}^{2}$, is the data fidelity term, which guarantees that the estimated C∘L closely matches the observed input *O*. The second part, ${∥\nabla L∥}_{p}$, is the flexible fractional-order norm with an adjustable parameter *p*, which is used to constrain the gradient of the scene transmission. This helps preserve the main structures while smoothing the textures. The third part, ${∥L-T_{0}∥}_{2}^{2}$, is the prior constraint that minimizes the $\ell_{2}$ distance between the transmission *L* and the initial parameter $T_{0}$:

(5) $$T_{0}=1-MO\left(\min_{c\in \left\{r,g,b\right\}}\left(\frac{I^{c}\left(x\right)}{A}\right)\right)$$

where $T_{0}$ is obtained via the morphological opening operator (MO) rather than the minimum operator, as suggested in [62]. The fourth part, ${∥W\circ \nabla C∥}_{1}$, enforces the scene radiance, which helps preserve the details and suppress the noise by designing a weight function that considers the local variances and gradients of the clear image. Mathematically, the weight matrix *W* is formulated as follows:

(6) $$W\left(x\right)=\frac{e^{-\lambda \cdot \left|G_{\sigma }\left(C^{2}\right)-{\left(G_{\sigma }\left(C\right)\right)}^{2}\right|}}{\max\left(\left|\nabla G_{\sigma }C\right|,\xi \right)}$$

where λ controls the smoothness of the inverted scene radiance *C*, $G_{\sigma }\left(\cdot \right)$ is the gradient filter with the standard deviation σ, and ξ is a small constant to avoid division by zero. Our designed weight function takes into account both the local variance and gradient information, which helps adaptively balance detail preservation and noise suppression. This design enables the model to better capture structural information while maintaining robustness in complex regions.

To optimize the designed mixed variational model, a block coordinate descent [63] was adopted to find the optimal solution to the non-convex objective function in Equation (4). Therefore, Equation (4) can be iteratively solved by decomposing it into two subproblems with respect to *L* and *C*, respectively, where each variable is updated alternately while keeping the other fixed. First, the terms associated with *L* are collected, yielding the following optimization problem for the *k*-th iteration:

(7) $$\underset{L}{\arg\min}{∥C_{k-1}\circ L-O∥}_{2}^{2}+\lambda_{1}{∥\nabla L∥}_{p}+\lambda_{2}{∥L-T_{0}∥}_{2}^{2}$$

Since $\ell_{p}$-norm may cause non-smooth optimization, an iteratively re-weighted least squares [64] strategy was employed, and Equation (7) can be rewritten as follows:

(8) $$\underset{L}{\arg\min}{∥C_{k-1}\circ L-O∥}_{2}^{2}+\lambda_{1}w_{L}{∥\nabla L∥}_{2}^{2}+\lambda_{2}{∥L-T_{0}∥}_{2}^{2}$$

where $w_{L}={\left(\max\left(\nabla L,\varepsilon \right)\right)}^{p-2}$.

Equation (8) contains only quadratic terms and, thus, corresponds to a convex problem with a closed-form global solution. Matrix notation was employed to rewrite Equation (8):

(9) $$\begin{array}{cc} \underset{L}{\arg\min}{\left(C_{k-1}\circ L-O\right)}^{T}\left(C_{k-1}\circ L-O\right) & +\lambda_{1}\left(L^{T}D_{x}^{T}W_{L_{x}}D_{x}L+L^{T}D_{y}^{T}W_{L_{y}}D_{y}L\right)\\ & +\lambda_{2}{\left(L-T_{0}\right)}^{T}\left(L-T_{0}\right) \end{array}$$

where $D_{x}$ and $D_{y}$ denote the Toeplitz matrices of the discrete forward difference gradient operators. $W_{L_{x}}$ and $W_{L_{y}}$ are diagonal matrices incorporating $w_{L_{x}}$ and $w_{L_{y}}$, respectively. By setting the first-order derivative of Equation (9) to zero, the closed-form solution of $L_{k}$ was obtained:

(10) $$L_{k}={\left(C_{k-1}^{T}C_{k-1}+\lambda_{1}\left(D_{x}^{T}W_{L_{x}}D_{x}+D_{y}^{T}W_{L_{y}}D_{y}\right)+\lambda_{2}1\right)}^{-1}\left(C_{k-1}^{T}O+\lambda_{2}T_{0}\right)$$

In order to avoid noise amplification in the sky regions, a lower bound of $t_{bound}$ was imposed on *L* during the iterative process.

Similarly, the second subproblem with respect to *C* can be formulated by considering only the terms related to *C*, as follows:

(11) $$\underset{C}{\arg\min}{∥C\circ L_{k}-O∥}_{2}^{2}+\lambda_{3}{∥W\circ \nabla C∥}_{1}$$

Furthermore, we reformulated Equation (11) as the following energy optimization function by applying the re-weighted least squares [64] strategy:

(12) $$\underset{C}{\arg\min}={∥C\circ L_{k}-O∥}_{2}^{2}+\lambda_{3}w_{C}{∥\nabla C∥}_{2}^{2}$$

where $w_{C}=W\circ {\left(\max\left(\nabla C,\varepsilon \right)\right)}^{-1}$.

Following the same approach as in Equation (9), Equation (12) is represented in matrix notation as follows:

(13) $$\underset{C}{\arg\min}{\left(C\circ L_{k}-O\right)}^{T}\left(C\circ L_{k}-O\right)+\lambda_{3}\left(C^{T}D_{x}^{T}W_{C_{x}}D_{x}C+C^{T}D_{y}^{T}W_{C_{y}}D_{y}C\right)$$

where $W_{C_{x}}$ and $W_{C_{y}}$ denote diagonal matrices that contain $w_{C_{x}}$ and $w_{C_{y}}$, respectively. The closed-form solution of Equation (13) is given by the following:

(14) $$C_{k}={\left(L_{k}^{T}L_{k}+\lambda_{3}\left(D_{x}^{T}W_{C_{x}}D_{x}+D_{y}^{T}W_{C_{y}}D_{y}\right)\right)}^{-1}\left(L_{k}^{T}O\right)$$

The updates of *L* and *C* are performed iteratively until Lk−Lk−1Lk−Lk−1Lk−1Lk−1<ε or Ck−Ck−1Ck−Ck−1Ck−1Ck−1<ε. For computational efficiency, a maximum iteration *K* is imposed, and the solver for the linear system is implemented using the preconditioned conjugate gradient (PCG) method [65]. The overall algorithm procedure is shown in Algorithm 1.
**Algorithm 1** Solution of mixed variational model (4).**Input:** *O*, parameters $\lambda_{1}$, $\lambda_{2}$, $\lambda_{3}$, and maximum number of iterations *K*.**Output:** *L* and *C***Initialization:** $L_{0}=O$, $C_{0}=1$1:**for** k=1 to *K* **do**2:    Update $w_{L}$.3:    Update $L_{k}$ using Equation (10).4:    Calculate *W* using Equation (6).5:    Update $w_{C}$.6:    Update $C_{k}$ using Equation (14).7:    **if** Lk−Lk−1Lk−Lk−1Lk−1Lk−1<ε or8:    Ck−Ck−1Ck−Ck−1Ck−1Ck−1<ε or k>K **then**9:        **break**10:    **end if**11:**end for**

## 4. Experimental Results and Discussion

In this section, we first describe the experimental settings to clarify the implementation details of our algorithm. We then present both visual and quantitative comparisons on real-world and synthetic hazy images to demonstrate its effectiveness. Furthermore, we analyze the the impact of the parameters and the computational complexity. Finally, we discuss its potential for generalization to broader applications and the limitations of the proposed algorithm.

### 4.1. Experimental Settings

All the experiments were carried out on a PC with an Intel(R) Core(TM) i5-8350U CPU (1.70 GHz) and 16 GB of RAM, using MATLAB R2019a. Unless otherwise stated, the empirical parameters in our proposed variational model were set as follows: $\lambda_{1}=0.01$, $\lambda_{2}=0.2$, $\lambda_{3}=0.0001$, and p=0.55. The stopping criterion ε was set to 0.01. For the weight computation, we set λ=100, the standard deviation σ=1, and the small constant ξ=0.001. The maximum number of iterations was limited to 20. For color hazy images, our algorithm was applied independently to each channel to ensure consistency across color components. Moreover, to enhance the overall brightness of the final dehazed result, a gamma correction (γ = 1/2.2) was applied.

### 4.2. Comparisons on Real-World Hazy Images

**Figure 2:**
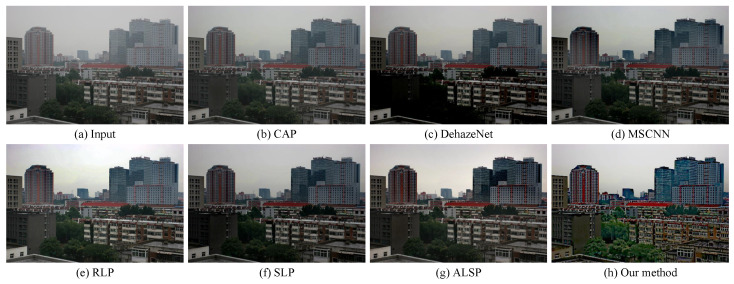
Visual comparison with state-of-the-art dehazing methods on a real-world hazy image collected from the project page (accessed on 15 November 2025) https://people.csail.mit.edu/kaiming/cvpr09/results.html of [9]. (**a**) A real-world hazy image. (**b**) CAP [10]. (**c**) DehazeNet [23]. (**d**) MSCNN [24]. (**e**) RLP [15]. (**f**) SLP [16]. (**g**) ALSP [18]. (**h**) Our method.

**Figure 3:**
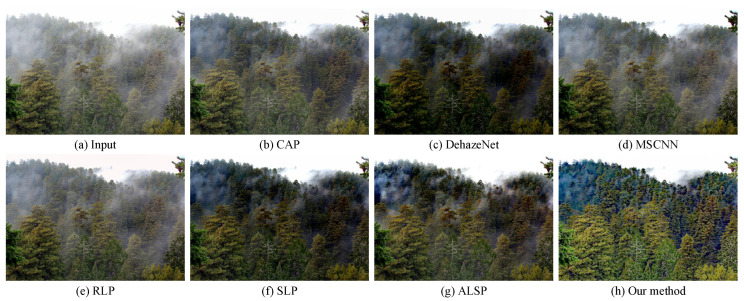
Visual comparison with state-of-the-art dehazing methods on a real-world hazy image collected from the project page (accessed on 15 November 2025) https://www.cs.huji.ac.il/w~raananf/projects/defog/ of [66]. (**a**) A real-world hazy image. (**b**) CAP [10]. (**c**) DehazeNet [23]. (**d**) MSCNN [24]. (**e**) RLP [15]. (**f**) SLP [16]. (**g**) ALSP [18]. (**h**) Our method.

**Figure 4:**
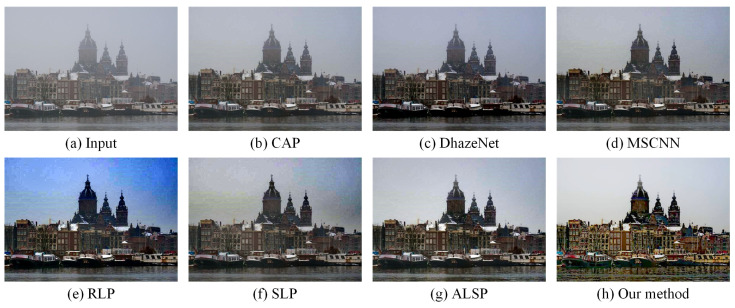
Visual comparison with state-of-the-art dehazing methods on a real-world hazy image from URHI dataset [67]. (**a**) A real-world hazy image. (**b**) CAP [10]. (**c**) DehazeNet [23]. (**d**) MSCNN [24]. (**e**) RLP [15]. (**f**) SLP [16]. (**g**) ALSP [18]. (**h**) Our method.

In order to prove the effectiveness of our proposed algorithm, we compared our approach with several state-of-the-art dehazing methods, including CAP (TIP’2015) [10], DehazeNet (TIP’2016) [23], MSCNN (ECCV’2016) [24], RLP (TIP’2021) [15], SLP (TIP’2023) [16], and ALSP (TIP’2025) [18], on the real-world hazy images. As shown in Figure 2, Figure 3 and Figure 4, CAP exhibited a limited dehazing capability, leaving a considerable amount of residual haze in the restored images. Both DehazeNet and MSCNN, trained on synthetic data, failed to generalize well to real-world hazy scenes, resulting in an unsatisfactory visual performance. RLP struggled to handle non-uniform haze, producing an uneven restoration across different regions, as illustrated in Figure 3. Although SLP performed well in removing haze from real-world images, the overall brightness of its results was relatively low, leading to visually dull appearances. ALSP effectively removed haze; however, it lacked sufficient edge enhancement, causing the restored images to appear less vivid and slightly desaturated. In contrast, our proposed algorithm not only removed haze effectively, but also enhanced the structural edges and restored natural color priors, achieving visually pleasing results with improved contrast, color fidelity, and detail preservation.

Furthermore, we conducted experiments on real-world datasets by randomly selecting 200 hazy images, including 100 from the Unannotated Real-World Hazy Images (URHI) dataset and 100 from the Real-World Task-Driven Testing Set (RTTS) of the RESIDE benchmark [67], denoted as the URHI test set and the RTTS test set, respectively. As ground-truth images are unavailable in these datasets, we employed four widely used no-reference image quality metrics, CNNIQA [68], MUSIQ [69], NIMA [70], and FADE [71], to evaluate the dehazing performance of state-of-the-art methods on real data. CNNIQA evaluates the perceptual image quality using a convolutional neural network. MUSIQ is a multi-scale image quality transformer that predicts the perceptual quality. NIMA employs a deep neural network to estimate the aesthetic and technical quality of images. FADE estimates the perceptual fog density. Higher scores for CNNIQA, NIMA, and MUSIQ indicate a better image quality, while a lower FADE score suggests a lower haze concentration. The results are presented in Table 1 and Table 2. As shown in Table 1, our algorithm achieved the highest scores for the CNNIQA and MUSIQ metrics, while securing second place for both the NIMA and FADE metrics. As shown in Table 2, our algorithm achieved the best performance for the CNNIQA metric and ranked within the top three for the MUSIQ, NIMA, and FADE metrics on real-world datasets. Although our algorithm performed well across all metrics, the slightly lower scores for NIMA and FADE can be attributed to their distinct evaluation criteria. NIMA emphasizes aesthetic quality, which involves subjective preferences that may not always align with our focus on structure preservation, while FADE prioritizes distortion reduction. Our algorithm balances between preserving fine details and minimizing distortions. Overall, these quantitative results highlight the superior generalization ability of our algorithm across different perceptual quality measures.

### 4.3. Comparisons on Simulated Hazy Images

**Figure 5:**
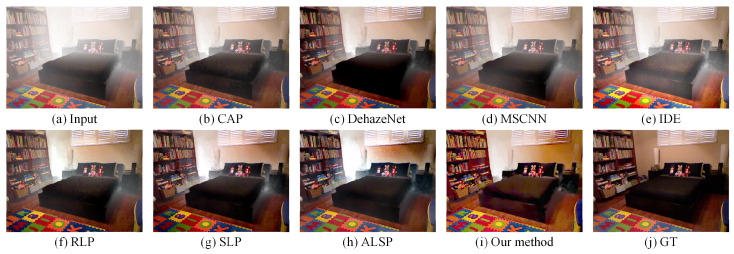
Visual comparison with state-of-the-art dehazing methods on a simulated hazy image collected from the D-HAZY dataset [73]. (**a**) A real-world hazy image. (**b**) CAP [10]. (**c**) DehazeNet [23]. (**d**) MSCNN [24]. (**e**) IDE [72]. (**f**) RLP [15]. (**g**) SLP [16]. (**h**) ALSP [18]. (**i**) Our method. (**j**) Groud truth.

**Figure 6:**
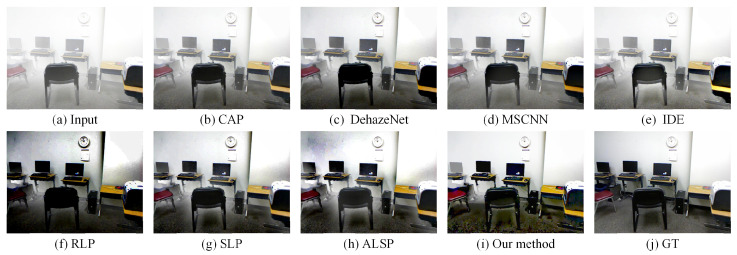
Visual comparison with state-of-the-art dehazing methods on a simulated hazy image collected from the D-HAZY dataset [73]. (**a**) A real-world hazy image. (**b**) CAP [10]. (**c**) DehazeNet [23]. (**d**) MSCNN [24]. (**e**) IDE [72]. (**f**) RLP [15]. (**g**) SLP [16]. (**h**) ALSP [18]. (**i**) Our method. (**j**) Groud truth.

To further assess the effectiveness of the proposed approach, comparative experiments were conducted on synthetic hazy images with several representative dehazing methods, including CAP (TIP’2015) [10], DehazeNet (TIP’2016) [23], MSCNN (ECCV’2016) [24], IDE (TIP’2021) [72], RLP (TIP’2021) [15], SLP (TIP’2023) [16], and ALSP (TIP’2025) [18], as presented in Figure 5 and Figure 6. The synthetic hazy images, collected from the D-HAZY dataset [73], were generated using the ASM, where the transmission map was computed from the depth map and an attenuation coefficient (β=1), with the atmospheric light set to [111]. For details on the construction of the synthetic hazy dataset and the parameters used, please refer to the article [73]. As observed, CAP, DehazeNet, MSCNN, and IDE exhibited a limited capability in haze removal, resulting in incomplete restoration with residual haze in several regions. RLP, SLP, and ALSP achieved more thorough dehazing; however, they tended to introduce halo artifacts around depth discontinuity edges, which degrade the structural consistency. Compared to these aforementioned methods, our proposed algorithm effectively removed haze while maintaining the edge integrity and natural color appearance, yielding restored results that are visually consistent and closely aligned with the ground truth.

In addition, to further validate the performance of our proposed approach, we conducted experiments on 100 synthetic hazy images randomly selected from the D-HAZY dataset [73]. We employed two widely used full-reference image quality metrics, PSNR and SSIM [74], along with two no-reference metrics, MUSIQ [69] and FADE [71], for a comprehensive evaluation. As summarized in Table 3, our proposed algorithm consistently ranked among the top two across all three metrics, demonstrating its strong capability in both haze removal and structural preservation. These results further confirm the effectiveness and robustness of our approach in restoring high-quality haze-free images.

### 4.4. Parameter Study

**Figure 7:**
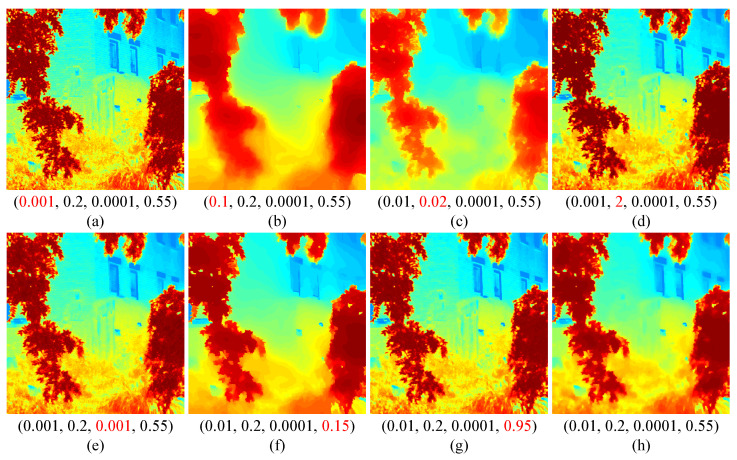
Effects of four parameters ($\lambda_{1}$, $\lambda_{2}$, $\lambda_{3}$, and *p*) for the scene transmission. Red values indicate deviations from the default settings.

We further analyzed the sensitivity of our proposed model to four key parameters, $\lambda_{1}$, $\lambda_{2}$, $\lambda_{3}$, and *p*, which jointly influence the accuracy of the estimated scene transmission. As shown in Figure 7, $\lambda_{1}$ controls the smoothness of the transmission map. When $\lambda_{1}$ is too small (e.g., $\lambda_{1}=0.001$), fine textures and noise are insufficiently smoothed (see Figure 7a), while an excessively large $\lambda_{1}$ (e.g., $\lambda_{1}=0.1$) leads to over-smoothing and structural distortion (see Figure 7b). The parameter $\lambda_{2}$ regulates the balance between texture preservation and structural consistency. A small $\lambda_{2}$ (e.g., $\lambda_{2}=0.02$) results in overly smoothed transmissions that obscure edges (see Figure 7c), whereas a large $\lambda_{2}$ (e.g., $\lambda_{2}=2$) introduces redundant details and weakens global coherence (see Figure 7d). When $\lambda_{3}$ was set to 0.001 (e.g., $\lambda_{3}=0.001$), subtle details could not be effectively smoothed, resulting in a visually uneven transmission estimation (see Figure 7e). The *p*-norm term controls the sparsity of the regularization. When *p* was too small (e.g., p=0.15), the transmission became over-smoothed, blurring structural features (see Figure 7f). Conversely, a large *p* (e.g., p=0.95) yielded insufficient smoothing, retaining undesirable texture variations (see Figure 7g). Overall, these results indicate that appropriate choices of $\lambda_{1}$, $\lambda_{2}$, $\lambda_{3}$, and *p* are essential for achieving an accurate and visually consistent transmission estimation (see Figure 7h). Accordingly, we empirically set $\lambda_{1}$, $\lambda_{2}$, $\lambda_{3}$, and *p* to 0.01, 0.2, 0.0001, and 0.55 in all experiments.

### 4.5. Computational Complexity

The computational complexity of the proposed dehazing method was further analyzed. For a color hazy image with a resolution of 600×450 (Figure 1), the processing time consumed approximately 24 s. The primary computational cost arose from the iterative optimization of the variational model, which is performed independently on each RGB channel. In particular, the preconditioned conjugate gradient (PCG) solver, which is used to handle the large sparse linear systems in each iteration, constitutes the major runtime bottleneck. Empirically, each PCG computation approximately takes about 0.2–0.4 s. Consequently, the total time spent in the PCG across all three channels accounted for approximately 90% of the overall runtime. It is worth noting that the current implementation is based on an unoptimized MATLAB prototype. Therefore, the runtime could be significantly reduced by employing a more efficient programming language (e.g., C/C++) or utilizing higher-performance hardware.

### 4.6. High-Level Computer Vision Tasks

**Figure 8:**
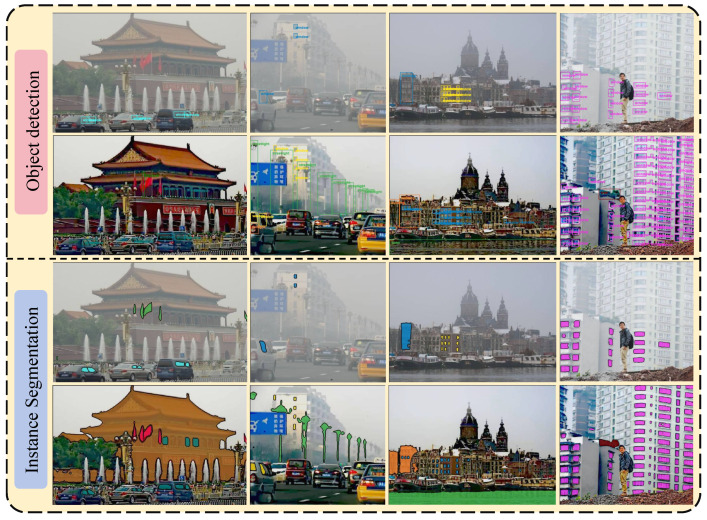
Performance improvement of high-level vision tasks.

To further assess the practical effectiveness of our proposed approach, we performed experiments using the unified vision model DINO-X [75] on two representative high-level vision tasks: object detection and instance segmentation. As illustrated in Figure 8, the input images substantially degraded the performance of these tasks, as object boundaries became ambiguous, small or distant objects were frequently missed, and the detection confidence was significantly reduced. In contrast, the images restored by our dehazing algorithm yielded clearer structural details and an enhanced contrast, enabling the more accurate localization and segmentation of targets. Therefore, our algorithm not only improves the visual quality, but also facilitates downstream vision applications under hazy conditions.

### 4.7. Generalization Applications

**Figure 9:**
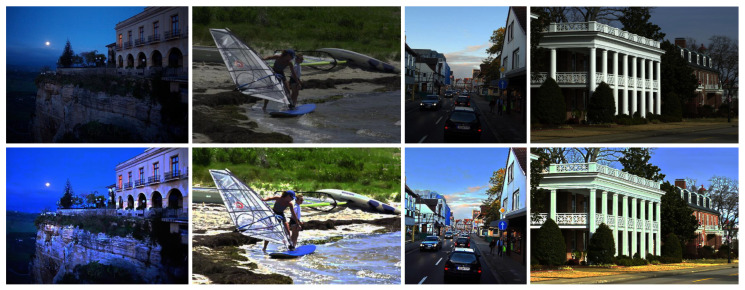
Generalization performance on low-light images.

**Figure 10:**
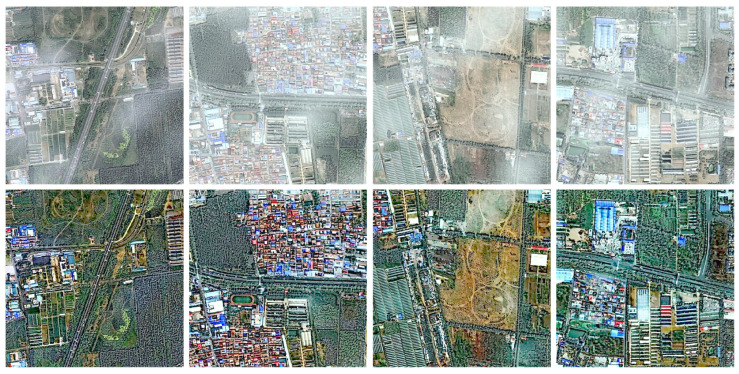
Generalization performance on remote sensing hazy images.

Our proposed algorithm demonstrates a remarkable generalization capability, allowing for its direct application to diverse low-level vision tasks without any modification or parameter adjustment. To evaluate the generalization performance of our algorithm, we used low-light images from published works [76,77,78] and remote sensing hazy images from the SateHaze1k dataset [79]. Our proposed model can be readily employed for low-light image enhancement by inverting the input image, processing the inverted version with our algorithm, and re-inverting the output. This simple, yet effective, strategy leverages the similarity between the degradation characteristics of inverted low-light images and hazy scenes. As illustrated in Figure 9, the enhanced results exhibit substantial improvements in brightness, contrast, and structural fidelity, producing visually natural and well-illuminated images. Furthermore, when applied to the remote-sensing hazy images in Figure 10, our algorithm successfully restored scene visibility and fine-grained textures, yielding clearer and more informative observations.

### 4.8. Limitations

**Figure 11:**
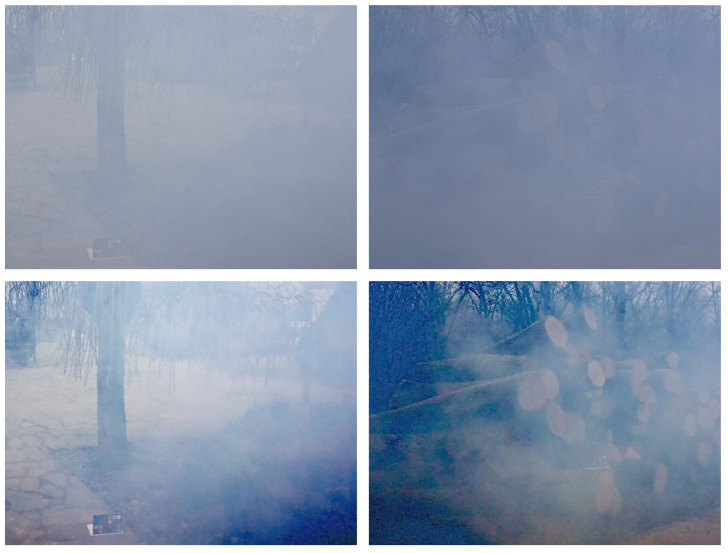
Limitations of the proposed algorithm in dense haze scenarios. The first row shows the input dense-haze images, and the second row presents the corresponding dehazed results.

Although our proposed framework achieved promising results in most haze scenarios and demonstrated a good generalization to low-light image enhancement and remote sensing dehazing tasks, there remain several limitations. As shown in Figure 11, we randomly selected challenging dense-haze images from the Dense-Haze dataset [80]. The dehazed results indicate that, under extremely dense haze conditions, our algorithm struggles to recover fine scene details and fails to fully restore the underlying structures. This limitation arises because, in dense haze, severe scattering and light attenuation lead to the significant loss of structural and color information, making an accurate estimation of the transmission map and scene radiance extremely difficult. In addition, our current algorithm has a relatively high computational complexity, which limits its applicability in real-time or resource-constrained scenarios.

In future work, we plan to enhance the adaptability of our model to extremely dense haze scenes by incorporating depth priors or physics-guided synthetic supervision. Meanwhile, we will explore lightweight network architectures and more efficient optimization strategies. Possible directions include employing unfolding networks to optimize the variational model, leveraging parallel or GPU-based acceleration to improve inference speed, and exploring fast approximate solvers to balance the computational efficiency and restoration performance.

### 4.9. Discussions

Our proposed haze removal method, which leveraged multiple variational constraints, demonstrated a competitive performance in both real-world and synthetic hazy scenes. As depicted in Figure 2, Figure 3, Figure 4, Figure 5 and Figure 6, the visual comparisons with representative state-of-the-art methods, such as CAP [10], DehazeNet [23], MSCNN [24], RLP [15], SLP [16], and ALSP [18], show that our approach consistently provides a superior image quality, with noticeable improvements in clarity and color restoration. In addition, the objective evaluations presented in Table 1, Table 2 and Table 3 further validate the effectiveness of our method, as it ranks among the top two in most metrics. Overall, these results demonstrate that our method demonstrated a competitive performance, with notable improvements in visual quality and ranking among the top two methods in most evaluation metrics compared to existing haze removal techniques.

## 5. Conclusions

In this paper, we proposed a novel variational-based dehazing framework for image quality improvements under haze conditions that simultaneously addresses transmission estimation and scene radiance recovery. Unlike conventional approaches, our method introduces a mixed regularization scheme that combines a flexible $\ell_{p}$ norm and an $\ell_{2}$ norm for transmission estimation, together with a weighted $\ell_{1}$ norm for scene radiance restoration. Furthermore, the adaptive weight function, designed to incorporate local variances and gradients, further strengthens the framework by effectively suppressing noise while maintaining structural fidelity. By integrating multiple regularization terms within a unified framework, the proposed algorithm achieves a balance between edge preservation, structural consistency, and noise suppression. Comprehensive experiments on both real-world and synthetic hazy images verified that the approach not only improves visual quality with sharper details and more natural colors, but also yields competitive results in terms of the objective metrics. In future work, we will focus on improving the computational efficiency to enable real-time applications, as well as extending the framework to more complex scenarios such as nighttime haze and dynamic scenes.

## Figures and Tables

**Table 1:** Quantitative comparisons on the UHRI test set. ↑ indicates that higher values are better, while ↓ indicates that lower values are better. The best and second-best results are highlighted using bold and underline, respectively.

Methods	Venue	URHI Test Set			
		CNNIQA↑	MUSIQ↑	NIMA↑	FADE↓
CAP [10]	TIP2015	0.6164	58.5949	4.5574	1.9631
DehazeNet [23]	TIP2016	0.6262	57.7627	4.7233	1.1125
MSCNN [24]	ECCV2016	0.6394	59.0667	4.6235	1.5314
RLP [15]	TIP2021	0.6682	59.2370	4.8978	0.7831
SLP [16]	TIP2023	0.6452	58.5328	4.8271	0.9496
ALSP [18]	TIP2025	0.6620	56.4242	**4.9617**	**0.4091**
Our method	-	**0.6793**	**59.4488**	4.9123	0.7583

**Table 2:** Quantitative comparisons on the RTTS test set. ↑ indicates that higher values are better, while ↓ indicates that lower values are better. The best and second-best results are highlighted using bold and underline, respectively.

Methods	Venue	RTTS Test Set			
		CNNIQA↑	MUSIQ↑	NIMA↑	FADE↓
CAP [10]	TIP2015	0.5954	56.8876	4.6774	1.8792
DehazeNet [23]	TIP2016	0.6039	56.4891	4.8311	1.1484
MSCNN [24]	ECCV2016	0.6191	57.5146	4.7402	1.3640
RLP [15]	TIP2021	0.6580	**58.4325**	**4.9433**	0.7502
SLP [16]	TIP2023	0.6304	57.1622	4.8620	0.8420
ALSP [18]	TIP2025	0.6405	55.9393	4.9329	**0.3926**
Our method	-	**0.6714**	58.0776	4.9121	0.7433

**Table 3:** Quantitative comparisons on a simulated hazy image dataset. ↑ indicates that higher values are better, while ↓ indicates that lower values are better. The best and second-best results are highlighted using bold and underline, respectively.

Methods	Venue	PSNR↑	SSIM ↑	MUSIQ↑	FADE↓
CAP [10]	TIP2015	10.4343	0.5927	39.9742	1.2507
DehazeNet [23]	TIP2016	12.2392	0.6111	41.7782	0.7251
MSCNN [24]	ECCV2016	9.9641	0.5828	42.0376	1.2075
IDE [72]	TIP2021	9.2873	0.5450	41.6226	0.9924
RLP [15]	TIP2021	11.8260	0.6139	**45.4642**	0.7474
SLP [16]	TIP2023	13.1661	**0.7135**	43.3794	0.5428
ALSP [18]	TIP2025	12.2068	0.6605	43.0191	**0.4892**
Our method	-	**13.7960**	0.6920	44.6151	0.5040

## Data Availability

The data are contained within the article.

## References

[1] Crameri F., Hason S. (2024). Navigating color integrity in data visualization. Patterns.

[2] Yang M. (2020). Investigating seasonal color change in the environment by color analysis and information visualization. Color Res. Appl..

[3] Ancuti C.O., Ancuti C. (2013). Single image dehazing by multi-scale fusion. IEEE Trans. Image Process..

[4] Li T., Liu Y., Luo S., Ren W., Lin W. (2025). Real-World Nighttime Dehazing via Score-Guided Multi-Scale Fusion and Dual-Channel Enhancement. IEEE Trans. Circuits Syst. Video Technol..

[5] Li T., Liu Y., Ren W., Shiri B., Lin W. (2025). Single Image Dehazing Using Fuzzy Region Segmentation and Haze Density Decomposition. IEEE Trans. Circuits Syst. Video Technol..

[6] McCartney E. (1976). Optics of the Atmosphere: Scattering by Molecules and Particles.

[7] Narasimhan S.G., Nayar S.K. Chromatic framework for vision in bad weather. Proceedings of the IEEE Conference on Computer Vision and Pattern Recognition 2000.

[8] Narasimhan S.G., Nayar S.K. (2002). Vision and the atmosphere. Int. J. Comput. Vis..

[9] He K., Sun J., Tang X. (2011). Single image haze removal using dark channel prior. IEEE Trans. Pattern Anal. Mach. Intell..

[10] Zhu Q., Mai J., Shao L. (2015). A fast single image haze removal algorithm using color attenuation prior. IEEE Trans. Image Process..

[11] Berman D., Treibitz T., Avidan S. (2020). Single image dehazing using haze-lines. IEEE Trans. Pattern Anal. Mach. Intell..

[12] Bui T.M., Kim W. (2018). Single image dehazing using color ellipsoid prior. IEEE Trans. Image Process..

[13] Singh D., Kumar V., Kaur M. (2019). Single image dehazing using gradient channel prior. Appl. Intell..

[14] Ju M., Ding C., Guo Y.J., Zhang D. (2019). IDGCP: Image dehazing based on gamma correction prior. IEEE Trans. Image Process..

[15] Ju M., Ding C., Guo C.A., Ren W., Tao D. (2021). IDRLP: Image dehazing using region line prior. IEEE Trans. Image Process..

[16] Ling P., Chen H., Tan X., Jin Y., Chen E. (2023). Single image dehazing using saturation line prior. IEEE Trans. Image Process..

[17] Liang S., Gao T., Chen T., Cheng P. (2024). A Remote Sensing Image Dehazing Method Based on Heterogeneous Priors. IEEE Trans. Geosci. Remote Sens..

[18] He L., Yi Z., Liu J., Chen C., Lu M., Chen Z. (2025). ALSP+: Fast Scene Recovery via Ambient Light Similarity Prior. IEEE Trans. Image Process..

[19] Cao Z.H., Liang Y.J., Deng L.J., Vivone G. (2025). An efficient image fusion network exploiting unifying language and mask guidance. IEEE Trans. Pattern Anal. Mach. Intell..

[20] Li L., Song S., Lv M., Jia Z., Ma H. (2025). Multi-Focus Image Fusion Based on Fractal Dimension and Parameter Adaptive Unit-Linking Dual-Channel PCNN in Curvelet Transform Domain. Fractal Fract..

[21] Krishnapriya S., Karuna Y. (2023). Pre-trained deep learning models for brain MRI image classification. Front. Hum. Neurosci..

[22] Nuanmeesri S. (2025). Enhanced hybrid attention deep learning for avocado ripeness classification on resource constrained devices. Sci. Rep..

[23] Cai B., Xu X., Jia K., Qing C., Tao D. (2016). DehazeNet: An end-to-end system for single image haze removal. IEEE Trans. Image Process..

[24] Ren W., Liu S., Zhang H., Pan J., Cao X., Yang M.H. Single image dehazing via multi-scale convolutional neural networks. Proceedings of the European Conference on Computer Vision (ECCV) 2016.

[25] Ren W., Pan J., Zhang H., Cao X., Yang M.H. (2020). Single image dehazing via multi-scale convolutional neural networks with holistic edges. Int. J. Comput. Vis..

[26] Li B., Peng X., Wang Z., Xu J., Feng D. Aod-net: All-in-one dehazing network. Proceedings of the IEEE International Conference on Computer Vision (ICCV) 2017.

[27] Deng Q., Huang Z., Tsai C.C., Lin C.W. Hardgan: A haze-aware representation distillation gan for single image dehazing. Proceedings of the European Conference on Computer Vision 2020.

[28] Zheng Y., Su J., Zhang S., Tao M., Wang L. (2022). Dehaze-AGGAN: Unpaired remote sensing image dehazing using enhanced attention-guide generative adversarial networks. IEEE Trans. Geosci. Remote Sens..

[29] Zhang S., Zhang X., Wan S., Ren W., Zhao L., Shen L. (2024). Generative adversarial and self-supervised dehazing network. IEEE Trans Ind. Inform..

[30] Zhang S., Zhang X., Shen L., Fan E. (2024). GAN-based dehazing network with knowledge transferring. Multimed. Tools Appl..

[31] Guo C.L., Yan Q., Anwar S., Cong R., Ren W., Li C. Image dehazing transformer with transmission-aware 3d position embedding. Proceedings of the IEEE/CVF Conference on Computer Vision and Pattern Recognition 2022.

[32] Liu Z., Lin Y., Cao Y., Hu H., Wei Y., Zhang Z., Lin S., Guo B. Swin transformer: Hierarchical vision transformer using shifted windows. Proceedings of the IEEE/CVF International Conference on Computer Vision 2021.

[33] Liu Y., Yan Z., Chen S., Ye T., Ren W., Chen E. Nighthazeformer: Single nighttime haze removal using prior query transformer. Proceedings of the 31st ACM International Conference on Multimedia 2023.

[34] Wang C., Pan J., Lin W., Dong J., Wang W., Wu X.M. Selfpromer: Self-prompt dehazing transformers with depth-consistency. Proceedings of the AAAI Conference on Artificial Intelligence 2024.

[35] Zhang S., Ren W., Tan X., Wang Z.J., Liu Y., Zhang J., Zhang X., Cao X. (2023). Semantic-aware dehazing network with adaptive feature fusion. IEEE Trans. Cybern..

[36] Zhang S., Zhang X., Ren W., Zhao L., Fan E., Huang F. (2025). Exploring Fuzzy Priors From Multi-Mapping GAN for Robust Image Dehazing. IEEE Trans. Fuzzy Syst..

[37] Zhang S., Zhang X., Shen L., Wan S., Ren W. (2025). Wavelet-Based Physically Guided Normalization Network for Real-time Traffic Dehazing. Pattern Recognit..

[38] Wang X., Yang G., Ye T., Liu Y. Dehaze-RetinexGAN: Real-World Image Dehazing via Retinex-based Generative Adversarial Network. Proceedings of the AAAI Conference on Artificial Intelligence 2025.

[39] Goodfellow I., Pouget-Abadie J., Mirza M., Xu B., Warde-Farley D., Ozair S., Courville A., Bengio Y. (2014). Generative adversarial nets. Adv. Neural Inf. Process. Syst..

[40] Vaswani A., Shazeer N., Parmar N., Uszkoreit J., Jones L., Gomez N.A., Kaiser L., Polosukhin I. (2017). Attention is all you need. Adv. Neural Inf. Process. Syst..

[41] Chen Z., Wang Y., Yang Y., Liu D. PSD: Principled synthetic-to-real dehazing guided by physical priors. Proceedings of the IEEE/CVF Conference on Computer Vision and Pattern Recognition (CVPR) 2021.

[42] Meng G., Wang Y., Duan J., Xiang S., Pan C. Efficient image dehazing with boundary constraint and contextual regularization. Proceedings of the IEEE International Conference on Computer Vision (ICCV) 2013.

[43] Galdran A., Vazquez-Corral J., Pardo D., Bertalmío M. A variational framework for single image dehazing. Proceedings of the Computer Vision-ECCV 2014 Workshops.

[44] Wang W., He C., Xia X.G. (2018). A constrained total variation model for single image dehazing. Pattern Recognit..

[45] Liu Q., Gao X., He L., Lu W. (2018). Single image dehazing with depth-aware non-local total variation regularization. IEEE Trans. Image Process..

[46] Liu Y., Shang J., Pan L., Wang A., Wang M. (2019). A unified variational model for single image dehazing. IEEE Access.

[47] Liu Y., Yan Z., Tan J., Li Y. (2023). Multi-purpose oriented single nighttime image haze removal based on unified variational retinex model. IEEE Trans. Circuits Syst. Video Technol..

[48] Jin Z., Ma Y., Min L., Zheng M. (2024). Variational image dehazing with a novel underwater dark channel prior. Inverse Probl. Imaging.

[49] Li C., Hu E., Zhang X., Zhou H., Xiong H., Liu Y. (2024). Visibility restoration for real-world hazy images via improved physical model and Gaussian total variation. Front. Comput. Sci..

[50] Liu Y., Wang X., Hu E., Wang A., Shiri B., Lin W. (2025). VNDHR: Variational Single Nighttime Image Dehazing for Enhancing Visibility in Intelligent Transportation Systems via Hybrid Regularization. IEEE Trans. Intell. Transp. Syst..

[51] Dwivedi P., Chakraborty S. (2023). Single image dehazing using extended local dark channel prior. Image Vis. Comput..

[52] Su L., Cui S., Zhang W. (2024). An Algorithm for Enhancing Low-Light Images at Sea Based on Improved Dark Channel Priors. J. Nav. Aviat. Univ..

[53] Song Y., He Z., Qian H., Du X. (2023). Vision transformers for single image dehazing. IEEE Trans. Image Process..

[54] Feng Y., Ma L., Meng X., Zhou F., Liu R., Su Z. (2024). Advancing real-world image dehazing: Perspective, modules, and training. IEEE Trans. Pattern Anal. Mach. Intell..

[55] Liu C., Ng M.K.P., Zeng T. (2018). Weighted variational model for selective image segmentation with application to medical images. Pattern Recognit..

[56] Zhao W., Wang W., Feng X., Han Y. (2022). A new variational method for selective segmentation of medical images. Signal Process..

[57] Du Y., Xu J., Qiu Q., Zhen X., Zhang L. Variational image deraining. Proceedings of the IEEE/CVF Winter Conference on Applications of Computer Vision 2020.

[58] Du Y., Xu J., Zhen X., Cheng M.M., Shao L. (2020). Conditional variational image deraining. IEEE Trans. Image Process..

[59] Hao S., Han X., Guo Y., Xu X., Wang M. (2020). Low-light image enhancement with semi-decoupled decomposition. IEEE Trans. Multimed..

[60] Fu G., Duan L., Xiao C. A Hybrid L2-LP variational model for single low-light image enhancement with bright channel prior. Proceedings of the 2019 IEEE International conference on image processing (ICIP).

[61] Hu E., Liu Y., Wang A., Shiri B., Ren W., Lin W. (2025). Low-Light Image Enhancement Using a Retinex-based Variational Model with Weighted L p Norm Constraint. IEEE Trans. Multimed..

[62] Zhou H., Zhao Z., Xiong H., Liu Y. (2022). A unified weighted variational model for simultaneously haze removal and noise suppression of hazy images. Displays.

[63] Tseng P. (2001). Convergence of a block coordinate descent method for nondifferentiable minimization. J. Optim. Theory Appl..

[64] Candes E.J., Wakin M.B., Boyd S.P. (2008). Enhancing sparsity by reweighted *ℓ*_1_ minimization. J. Fourier Anal. Appl..

[65] Barrett R., Berry M., Chan T.F., Demmel J., Donato J., Dongarra J., Eijkhout V., Pozo R., Romine C., Van der Vorst H. (1994). Templates for the Solution of Linear Systems: Building Blocks for Iterative Methods.

[66] Fattal R. (2008). Single image dehazing. ACM Trans. Graph..

[67] Li B., Ren W., Fu D., Tao D., Feng D., Zeng W., Wang Z. (2019). Benchmarking single-image dehazing and beyond. IEEE Trans. Image Process..

[68] Kang L., Ye P., Li Y., Doermann D. Convolutional neural networks for no-reference image quality assessment. Proceedings of the IEEE Conference on Computer Vision and Pattern Recognition 2014.

[69] Ke J., Wang Q., Wang Y., Milanfar P., Yang F. Musiq: Multi-scale image quality transformer. Proceedings of the IEEE/CVF International Conference on Computer Vision 2021.

[70] Talebi H., Milanfar P. (2018). NIMA: Neural image assessment. IEEE Trans. Image Process..

[71] Choi L.K., You J., Bovik A.C. (2015). Referenceless prediction of perceptual fog density and perceptual image defogging. IEEE Trans. Image Process..

[72] Ju M., Ding C., Ren W., Yang Y., Zhang D., Guo Y.J. (2021). IDE: Image dehazing and exposure using an enhanced atmospheric scattering model. IEEE Trans. Image Process..

[73] Ancuti C., Ancuti C.O., De Vleeschouwer C. D-HAZY: A dataset to evaluate quantitatively dehazing algorithms. Proceedings of the 2016 IEEE International Conference on Image Processing (ICIP).

[74] Wang Z., Bovik A.C., Sheikh H.R., Simoncelli E.P. (2004). Image quality assessment: From error visibility to structural similarity. IEEE Trans. Image Process..

[75] Ren T., Chen Y., Jiang Q., Zeng Z., Xiong Y., Liu W., Ma Z., Shen J., Gao Y., Jiang X. (2024). Dino-x: A unified vision model for open-world object detection and understanding. arXiv.

[76] Fu X., Liao Y., Zeng D., Huang Y., Zhang X., Ding X. (2015). A probabilistic method for image enhancement with simultaneous illumination and reflectance estimation. IEEE Trans. Image Process..

[77] Guo X., Li Y., Ling H. (2017). LIME: Low-light image enhancement via illumination map estimation. IEEE Trans. Image Process..

[78] Fu X., Zeng D., Huang Y., Zhang X., Ding X. A weighted variational model for simultaneous reflectance and illumination estimation. Proceedings of the IEEE/CVF Conference on Computer Vision and Pattern Recognition (CVPR) 2016.

[79] Huang B., Zhi L., Yang C., Sun F., Song Y. Single satellite optical imagery dehazing using SAR image prior based on conditional generative adversarial networks. Proceedings of the IEEE/CVF Winter Conference on Applications of Computer Vision 2020.

[80] Ancuti C.O., Ancuti C., Sbert M., Timofte R. Dense haze: A benchmark for image dehazing with dense-haze and haze-free images. Proceedings of the IEEE International Conference on Image Processing (ICIP) 2019.

